# Effects of game-based physical education program on enjoyment in children and adolescents: a systematic review and meta-analysis

**DOI:** 10.1186/s12889-024-18043-6

**Published:** 2024-02-19

**Authors:** Weilong Mo, Jamalsafri Bin Saibon, Yaming LI, Jiequan Li, Yanwu He

**Affiliations:** 1 Malaysia Postgraduate Department, School of Educational Studies, Universiti Sains Malaysia, 11800 Penang, Malaysia; 2 Zhaoqing College, Zhaoqing, 526061 China; 3 Dinghu First Experimental School, Zhaoqing, 526070 China; 4 Huaqiao Junior High School, Zhaoqing, 526108 China

**Keywords:** Enjoyment, Game-based, Physical education, Children and adolescents, Meta-analysis

## Abstract

**Objective:**

The objective of this study was to conduct a systematic review to summarize and assess the advancements lately made on the enjoyable impacts of game-based physical education interventions on children and adolescents. Additionally, it attempted to identify the effects and variables influencing the enjoyable outcomes of children and adolescents’ engagement in physical education games, through meta-analysis.

**Methods:**

This study involves a comprehensive search of different databases like Web of Science, PubMed, Embase, EBSCOhost, Cochrane, and Scopus. Specific criteria are established for the selection process to make sure the relevant literature included. The quality assessment of the included researches is conducted based on the guidelines outlined in the Cochrane 5.1 handbook. Review Manager 5.3 software is employed to synthesis the effect sizes. Additionally, bias is assessed using funnel plots, and to identify potential sources of heterogeneity, subgroup analyses are performed.

**Results:**

A total of 1907 academic papers, out of which 2 articles were identified via other data sources. The present study examined the impact of a pedagogical intervention involving physical education games on the enjoyment experienced by children and adolescents. The results indicated a significant positive effect (MD = 0.53, 95%CI:[0.27,0.79], *P* < 0.05) of this intervention on enjoyment. Subgroup analyses further revealed that both boys (MD = 0.31, 95%CI:[0.13,0.50], *P* < 0.05) and girls (MD = 0.28, 95%CI:[0.05,0.51], *P* < 0.05) experienced increased pleasure compared to traditional physical education. Additionally, children under 12 years of age (MD = 0.41, 95%CI:[0.17,0.64], *P* < 0.05) benefited from sessions lasting at least 30 minutes or more per session (MD = 0.40, 95%CI:[0.19,0.60], *P* < 0.05), occurring 1 to 3 times per week (MD = 0.28, 95%CI:[0.16,0.40], *P* < 0.05), and lasting for more than 3 weeks (MD = 0.81, 95%CI:[0.29,1.34], *P* < 0.05). These findings suggest that the implementation of physical education games can be an effective approach to teaching this subject.

**Conclusions:**

1) Interventions using physical games have been shown to yield beneficial outcomes in terms of enhancing the enjoyment experienced by children and adolescents. 2) The effectiveness of treatments aimed at promoting enjoyment among children and adolescents is influenced by several aspects, including gender, age, duration and frequency of physical activity, as well as the specific cycle of activity used.

## Introduction

Enjoyment is a subjective experience with pleasant emotions, such as pleasure, like, and fun [1]. Children and adolescents are naturally motivated by enjoyable experiences during the learning process, which enhances their academic achievement, involvement and effort in learning [2–4], this, in turn, leads to more effective and long-lasting learning [5, 6]. In contrast, falling enjoyment can diminish their interest and engagement. Therefore, the cultivation of enjoyable feelings in children and adolescents has a crucial role in enhancing educational achievements.

Studies proved that physical education has a beneficial influence on the psychological and physical health of children and adolescents [7, 8], as well as on the prevention of disease problems [9]. The influence of physical education on the enjoyment of children and adolescents, particularly in relation to emotions, has clear benefits [10]. Physical education activities for children and adolescents not only have the power to enhance individual happiness, but also foster a positive team atmosphere and promote collaboration and socialization [11]. Thus, it is essential to explore the positive impact of these activities on enjoyment and mental health.

Compared with traditional physical education, physical education games have several advantages. The implementation of physical play interventions has the potential to facilitate the acquisition of knowledge and skills among children and adolescents [12, 13], enabling them to get enjoyment from the process of learning. A combination of entertainment components into traditional physical education (PE) is effective in motivating non-athletic students to actively engage in PE lessons, which cannot be achieved through organized sports [14]. Liao et al. [15] further explain that games not only enhance students’ satisfaction with PE lessons, but also facilitate skill development, create a relaxed play environment, foster interpersonal interactions, and offer opportunities for cooperation and socialization.

Hence, the implementation of physical games teaching offers a new and innovative approach within the context of traditional physical education classes [16]. This approach to learning is not only pleasurable for students, but also meets their requirement for social and physical engagement in the educational process and, most notably, contributes to a key part in sustaining the involvement of children and adolescents in physical education and sports [17].

Through a comprehensive analysis of 16 academic studies, it has been noticed that further research is needed regarding the efficacy of applying physical education games to enhance enjoyment in children and adolescents. Since the majority of studies show a beneficial effect on enjoyment [1, 18–26]. However, five studies remain uncertain about the impact of these games [14, 27–30], and there is even a case where teaching with games seems to reduce students’ enjoyment [27]. Currently, there is not enough comprehensive analysis on how physical game teaching impacts the enjoyable feelings of children and adolescents, and also on the potential factors that may influence those effects (such as gender, age, and duration of the interventions).

The aim of this study was to explore the following topics with a meta-analysis: 1) whether teaching games in physical education has a beneficial influence on enjoyment experienced by children and adolescents, and 2) how other essential elements mitigate the influence of games teaching in physical education on enjoyment experienced by children and adolescents. Due to inconsistent findings from earlier research, there is no consensus on the link of physical games for enjoyment in children and adolescents; yet there is optimism that teaching physical play may have a favorable impact on enjoyment.

## Methods

### Search strategy and standards for selection

This research is conducted under the guidance of Cochrane Handbook for the Systematic Review of Interventions [31] and the PRISMA Statement Specification for Systematic Review and Meta-analysis [32].

This research explores six databases, namely Web of Science, PubMed, Embase, EBSCOhost, Scopus and Cochrane. PubMed is primarily adopted to identify medical terms (Mesh). The search period spans from the initiation of database collection until July 25, 2023. Both subject terms and the free word approach are included in the search. The following table consists of many columns. This study firstly focuses on the research object and then emphasizes the intervention strategy. In the third line, the result index is built by connecting the search words using the logical operator “or” inside each group of search terms. Additionally, the search phrases are linked by “or” between each set of search terms. Table 1 displays the whole search words used in the six databases.

**Table 1:** Search terms

Search term classification	Search term content
Subject of intervention	Students (Mesh terms), Student; School Enrollment; Enrollment, School; Enrollments, School; School Enrollments; Adolescent (Mesh terms); Adolescents; Adolescence; Teens; Teen; Teenagers; Teenager; Youth; Youths; Adolescents, Female; Adolescent, Female; Female Adolescent. Female Adolescents; Adolescents, Male; Adolescent, Male; Male Adolescent; Male Adolescents, Child (Mesh terms), children; juvenile; school-aged children
Type of intervention	Game; games; Physical Education and Training (Mesh terms); Physical Education, Training; Physical Education; Education, Physical;Teaching games;Teaching sports;Sport pedagogy
Ending indicators	Pleasure (Mesh terms); Gratification; Enjoyment; Satisfaction; Delight; Fun; Diversion; Delectation

### Criteria for inclusion and exclusion

Research that meet the following requirements are selected for systematic evaluation: (1) the intervention modality is physical game teaching; (2) the subjects are children and adolescents (3–18 years old); (3) the outcome indicator is the inclusion of enjoyment related MeSH and Entry Terms; (4) the use of a control group; and (5) the articles are written in English.

Researches reach the following standards are omitted from systematic evaluation: (1) the intervention method is not physical game teaching; (2) the experimental subjects are infants, adults, animals, and specific populations (psychiatric patients), etc.; (3) the outcome indicator is the absence of pleasure-related subject terms and free words; (4) no control group is included; (5) the articles are written in other non-English languages; (6) review article; and (7) conference articles.

### Screening process

**Fig. 1:**
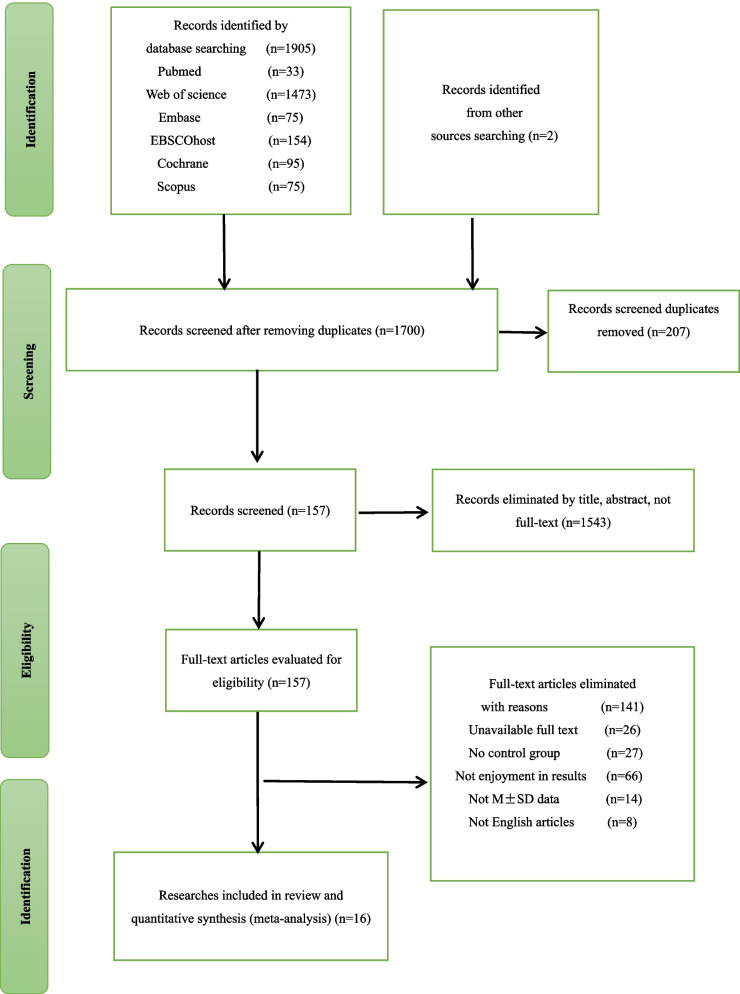
Flowchart for inclusion and exclusion of studies

Upload the relevant literature to Endnote (version X9) for organization. Following this, duplicate results are screened by two authors (MWL and LYM) independently. The screening process include reviewing titles, review articles, conference papers, and animal experiments. Read the abstracts to exclude articles that fail to meet such criteria as study subjects or interventions. Finally, read the full text of selected articles to exclude those that are inaccessible, non-English and does not provide end point indicators. The process involves an initial screening of eligible articles, a discussion of any discrepancy and reaching a consensus with the third author (LJQ). Ultimately, 16 articles are selected for the systematic analysis. Detailed information about these steps are presented in the PRISMA flowchart (refer to Fig. 1).

### Extraction of data and quality evaluation

Three authors (MWL, LYM, and LJQ) extract data from eligible papers in an impartial manner. Any divergence is resolved through discussion until an agreement is reached. The extracted information is then placed in the publications respectively [1]. the extracted information primarily includes the name of the first author and the publication year [2]. the subjects’ features encompass the total sample size, age, and gender [3]. detailed data, like duration, frequency, and cycle, about the teaching process of physical education and sport games are included [4]. the intervention tool employed in the study is a questionnaire or scale designed to measure the degree of pleasure, satisfaction and motivation before and after physical education [5]. the intervention items utilized in the study are game items specifically employed for physical education [6]. the outcome indicators encompass various factors, including the level of pleasure, satisfaction, and motivation before and after physical education and sport activities. Pleasure may be defined as the state of experiencing gratification, enjoyment, satisfaction, delight, or fun [7]. the writers emphasize the importance of significant results.

**Fig. 2:**
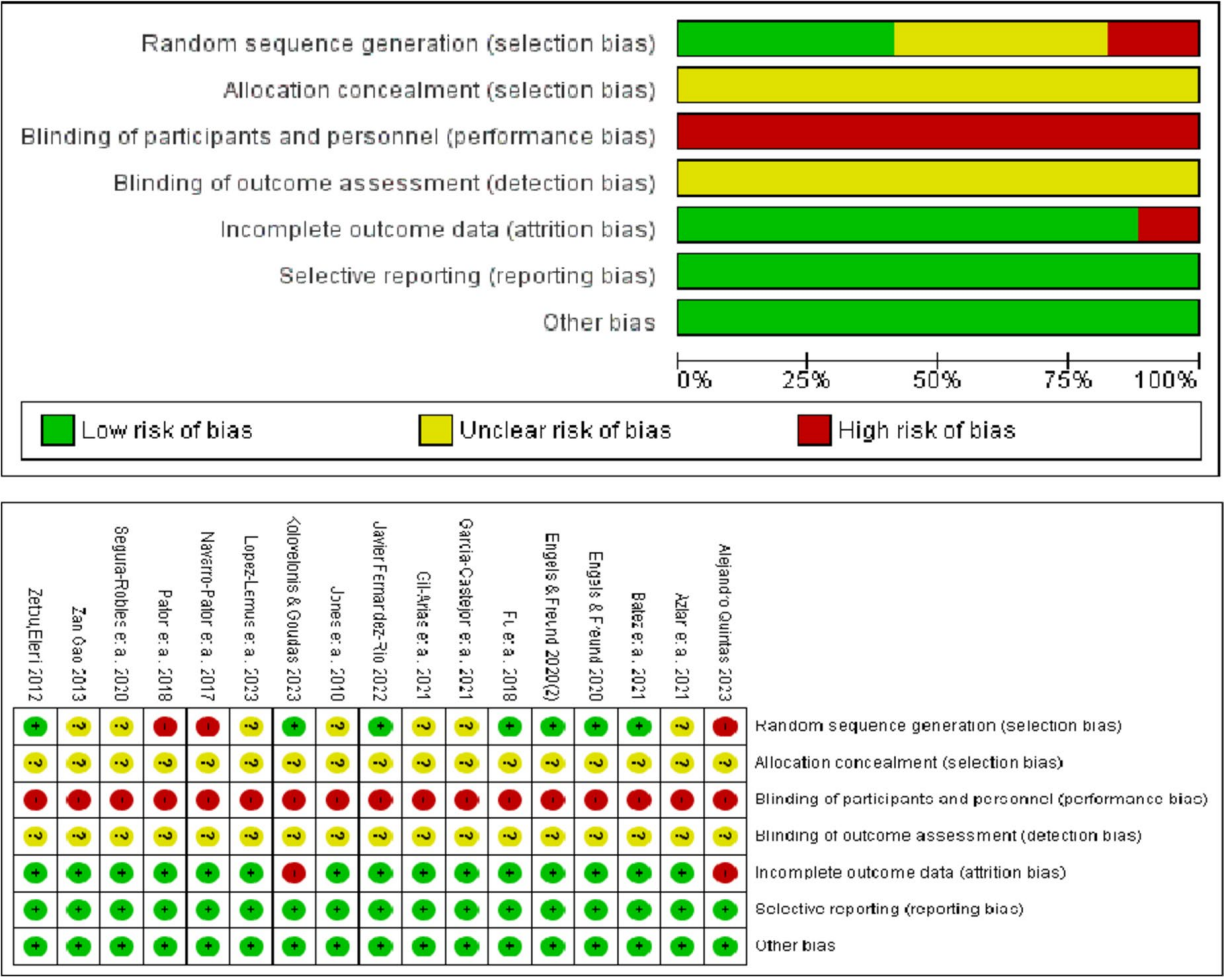
Cochrane risk of bias evaluation

The Cochrane 5.1 handbook is applied to assess the quality of bias. The evaluation includes one aspect, namely the random allocation procedure and the concealing of allocation schemes. In this research, the evaluation criteria are: 3) participant blinding and outcome assessment, 4) complete outcome data, 5) selective report findings, and 6) bias from other sources. Each criterion is assessed as having either a low risk (an indication for meeting the criterion), a high risk (an indication for not satisfying the criterion), or a medium risk (if not mentioned), with a note explaining the reason for this assessment. Two researchers assesses the article quality independently. Any divergence in their evaluations are solved by discussing with another author. Figure 2 shows the evaluation findings and the detailed information. A sensitivity analysis is conducted whereby each article is systematically removed one at a time. The analysis reveals that the findings are mostly unchanged, which suggests that the results are robust and reliable.

### Statistical analysis

Review Manager 5.3, a statistical software, is applied to merge effect sizes and assess for bias. In this analysis, the indicator continuous variable is incorporated, making the results presented as mean ± standard deviation (Mean ± SD). The I^2^ and Q tests are adopted to evaluate heterogeneity between studies. The fixed-effects model is applied as the I^2^ < 50% or *p* > 0.1, indicating the studies lack statistical heterogeneity. In contrast, a random-effects model is adopted to evaluate publication bias using funnel plots and to examine the reliability of the findings.

## Results

### Search results

A comprehensive search for 1907 articles in total is conducted. The databases for search are Web of Science (1473 articles), PubMed (33 articles), Embase (75 articles), EBSCO host (154 articles), Cochrane (95 articles), and Scopus (75 articles). All identified articles are uploaded to Endnote (version X9), a reference management software. By examining the article titles, a total of 207 duplicate items are eliminated from further analysis. The articles are uploaded to Endnote (version X9), and after examining the titles, a total of 207 duplicates are removed. The dataset comprises 321 conference papers, 44 review articles, 31 articles with inconsistent subjects, 1 article applying inconsistent measurement tools, 94 articles employing inconsistent interventions, 66 articles featuring different endpoints as determined after reading the full text. 14 articles presenting results deviate from the mean ± standard deviation format. A total of 27 publications without a control group are identified, and 8 articles written in non-English languages are excluded. Additionally, the full texts of 26 articles are incomplete or unavailable. Hence, a final set of 16 eligible articles is included in the meta-analysis.

### Basic features of the included articles

The analysis encompasses 16 articles, which collectively examines 17 studies. The total sample size has 2181 participants, with 1139 individuals assigned to the experimental group and 1042 individuals assigned to the control group. There were 1096 male participants and 1048 female ones. The age of the samples ranges from 4.9 to 15.62 years. The intervention duration covers from 15 to 90 minutes, with frequencies from 1 to 9 times each week. The intervention cycles span from 2 to 14 weeks. The interventions are concentrated on sports and games programs. The assessed outcome indicators include enjoyment and satisfaction, intervention instruments and major findings. See Table 2 for detailed information.

**Table 2:** Summary of features of included interventions

First author Year	Sample size characteristics	Age	Duration	Frequency	Cyclicality	Intervention instruments	Intervention programmes	Ending indicators	Key findings
Azlan et al. 2021 [14]	Students 56 = boys (57%; *n* = 32) and girls (43%; *n* = 24)	13.5 ± 0.5 years old	40 min	One sessions /week	2 weeks	A simple questionnaire of acceptance of playing TG and PE lesson activities was developed based on Wasowicz.and Zeng, Hipscher	Galah Panjang (Long Pole) and Baling Selipar (Throwing Slipper)	Enjoyment	The mean acceptance scores showed students preferred playing TG (19.29 ± 4.21; *p* < 0.00) over free-play PE lesson (17.59 ± 3.13).
Engels & Freund 2020 [28]	Students 143 = boys (56.6%; *n* = 81) and girls (43.4%; *n* = 6)	12.50 ± 0.71 years old	15 min	One sessions /week	7 weeks	Questionnaire for the Assessment of Enjoyment in Physical Education	The Magical Hoops、Mississipp, Chimney Sweep, Blanket Turnaround, Change of Places, Space in the Smallest Hut, Human Knot, 7 People with 4 Feet, Siamese Soccer, Transportation on a Conveyor Belt, Save me if you can!	Enjoyment	cooperative games led to a higher perceived enjoyment in physical education classes (F [1] = 3.49, *p* = .063, ηp 2 = .012), increased the feeling of how strong students felt related to each other (F [1] = 4.38, *p* = .037, ηp 2 = .016)
Garcia-Castejon et al. 2021 [29]	Students 99 = boys (48.5%; *n* = 48) and girls (51.5%; *n* = 51)	12.63 ± 0.72 years old	50 min	two sessions /week	11 weeks	PNSE Basic Psychological Needs Questionnaire. Sport Satisfaction Instrument SSI Questionnaire	basketball, futsal, and volleyball	Enjoyment	the hybridization between the TPSR and TGfU model is presented as an effective alternative to be applied in the educational context with the aim of improving young peoples’ intention to be physically active and psychological variables, such as motivation, responsibility, and enjoyment, in physical education classes
Jones et al. 2020 [24]	Students 202 = boys (49.0%; *n* = 99) and girls (51.0%; *n* = 103)	11–14 years old	No report	No report	6 weeks	Intrinsic Motivation Inventory (IMI)	an invasion games unit	Enjoyment	Two way (group x gender) ANCOVA adjusted for the baseline subscale identified: significant main effects for teaching approach for interest & enjoyment (F(1,197) = 241.74, *p* < 0.001, eta squared = .55)
Navarro-Paton et al. 2017 [25]	Students 104 = boys (56.7%; *n* = 59) and girls (43.3%, *n* = 5)	10.29 ± 0.62 years old	No report	two sessions /week	3 weeks	scale of multidimensional measure referring to enjoyment, competence, relationship, autonomy, demotivation, intrinsic and extrinsic motivation	playing cooperative games	Enjoyment	in the experimental group, the scores of the variables autonomous motivation (*p* = 0.000), SDI (*p* = 0.005), relationship (*p* = 0.004), autonomy (*p* = 0.017), PMI (*p* = 0.004), enjoyment (*p* = 0.001), personal responsibility (*p* = 0.015), social responsibility (*p* = 0.001) and IPA (*p* = 0.000) increased
Paton et al. 2018 [30]	Students 98 = boys (54.1%; *n* = 53) and girls (45.9%; *n* = 45)	10.40 ± 0.50 years old	No report	two sessions /week	3 weeks	a multidimensional Scale was used according to enjoyment, competence, relation with others, autonomy, demotivation, intrinsic motivation and extrinsic motivation	competitive games	Enjoyment	the didactic unit based on the competitive games in the EF classes caused significant improvements in the different dimensions
Segura-Robles et al. 2020 [18]	Students 64 = boys(43.75%; *n* = 28) and girls(56.25%; *n* = 36)	15 ± 1.62 years old	No report	No report	5 weeks	Basic Psychological Needs in Exercise Scale; Sport Motivation; Scale; Sport Satisfaction Instrument;	a flipped and gamified program	Enjoyment	students’ satisfaction, enjoyment, and intrinsic motivation have improved based on the interaction with gamification and flipped learning
Batez et al. 2021 [23]	Students 54 = boys(66.7%; *n* = 36) and girls(33.3%; *n* = 18)	15.62 ± 0.65 years old	45 min	two sessions /week	6 weeks	Sports Enjoyment Scale	mini-volleyball	Enjoyment	The EXP group showed significantly better results for enjoyment compared to the CON group (p ≤ 0.05)
Lopez-Lemus et al. 2023 [21]	Students 137 = boys(54.7%; *n* = 75) and girls(45.3%; *n* = 62)	14.18 ± 0.83 years old	55 min	two sessions /week	12 weeks	Game Performance Assessment Instrument (GPAI);Enjoyment and Perceived Competence;Intention to Be Physically Active	handball via	Enjoyment	the application of hybrid models SE/TGfU could increase and help facilitate students’ game involvement and game performance, enjoyment, perceived competence and intention to be physically active
Fu et al. 2018 [33]	Students 65 = boys(52.30%; *n* = 34) and girls(47.7%; *n* = 31)	4.9 ± 0.7 years old	30 min	five sessions /week	12 weeks	Gross Motor Development-Edition 3 (TGMD-3);Intrinsic Motivational Inventory	GoNoodle;Adventure to Fitness;Cosmic Kids Yoga	Enjoyment	There were also significant positive and weak correlations between enjoyment and the object control subtest and TGMD-3 total score (*P* < 0.05). Moderate-to-strong correlations were found among subtest scores and the total score of the TGMD-3
Kolovelonis & Goudas 2023 [20]	Students 102 = boys(54.9%; *n* = 56) and girls(45.1%; *n* = 46)	10.13 ± 0.57 years old	45 min	No report	No report	The Design Fluency Test;Situational Interest Scale	hop-pop-tag;modified crazy traffic lights;the mirror; the maps	Enjoyment	participated in cognitively challenging physical activity games students compared to participated in activities for developing their health-related fitness students scored higher in total interest (*p* = 0.004, d = 0.71) and in instant enjoyment (*p* < 0.001, d = 0.89).
Gil-Arias et al. 2020 [19]	Students 292 = boys(52.1%; *n* = 152) and girls(47.9%; *n* = 140)	10.41 ± 0.49 years old	50 min	two sessions /week	8 weeks	BPNs in exercise scale;Physical Activity Class Satisfaction Questionnaire	basketball	Enjoyment	regardless of students gender and/or content focus of the unit and have opportunities to increase their engagement, enjoyment, and social interactions within physical education lessons.
Fernandez-Rio et al. 2022 [22]	Students 54 = boys(51.9%; *n* = 28) and girls(48.1%; *n* = 26)	14 ± 0.1 years old	90 min	a sessions /week	9 weeks	satisfaction and frustration scale	jump Ropes;Double Dutch rope jumping	satisfaction	showed signifi-cant mean increases in the experimental group in all the variables: intrinsic motivation (*P* = 0.001, d = 1.48), autonomy satisfaction (*P* = 0.001, d = 0.65), competence satisfaction (*P* = 0.003, d = 0.77), relatedness satisfaction (*P* = 0.010, d = 1.01), intention to be physically active (*P* = 0.001, d = 0.75) and only autonomy satisfaction (*P* = 0.027, d = 0.65) in the comparison group
Gao et al. 2013 [1]	Students 53 = boys(45.3%; *n* = 24) and girls(54.7%; *n* = 29)	10.3 years old	30 min	No report	3 weeks	Enjoyment-Competence scale	Dance Dance Revolution	Enjoyment	Children spent more moderate-to-vigorous PA (MVPA) time (*p* < 0.01, h2 ¼ 0.49) in aerobic dance than DDR. Additionally, children reported significantly higher self-efficacy (*p* < 0.001, h2 ¼ 0.28) and enjoyment (*p* < 0.01, h2 ¼ 0.18) in DDR than in aerobic dance.
Quintas & Bustamante 2023 [27]	Students 417 = boys(46.8%; *n* = 195) and girls(53.2%; *n* = 222)	11.1 ± 1.7 years old	45 min	three sessions /week	4 weeks	Sport Satisfaction Instrument	didactic dance (added a gamified climate in class and used an exergame)	Enjoyment	the gamified exergaming education intervention would produce more enjoyment in students than the non-gamified and non-exergaming intervention. An interaction effect was found on enjoyment (F(1) = 7.56, *p* = .006, h2 *p* = .02)
Zetou et al. 2012 [26]	Students 62 = boys(48.4%; *n* = 30) and girls(51.6%; *n* = 32)	11.13 ± .33 years old	20 min	nine sessions /week	4 weeks	Evaluation of satisfaction(a six-item scale)	Play and Stay program (tennis)	Satisfaction	students will be motivated to become more involved in tennis as a sport, since enjoyment and satisfaction stimulate interest in participation

All figures in the table (age) column are means ± standard deviation

Sample size characterisation figures for tables are processed through a uniform format

*TG* Traditional games, *PE* Physical education, *PNSE* Psychological Needs in Exercise Scale, *TGfU* Teaching Games for Understanding, *ANCOVA* Analysis of covariance, *EF* Physical Education, *EXP* Experimental, *CON* Control, *SE/TGfU* Sport Education/Teaching for understanding, *TGMD-3* Test for Gross Motor Development-Edition 3, *MVPA* Moderate to vigorous physical activity, *BPNs* Basic psychological needs, *DDR* Dance Dance Revolution

### Quality assessment

This research examines the literature about the random assignment process and specifically focuses on six studies that meet the inclusion criteria [20, 22, 23, 26, 28, 33]. The remaining research do not provide details about the randomization process. None of the 17 studies mention whether the allocation is concealed or not in the allocation scheme concealment. In terms of blinding, researchers on the subjects and inform them about the tests. Thus, the subjects are not blinded. Consequently, all 17 studies are deemed to have a high risk. The evaluation of the findings is featured with uncertainty. Two studies had a high incidence of staff turnover as for the completeness of the outcome data [20, 27]. None of the 15 studies shows any subject or data loss, and all of them are considered to have low risk. The included studies show no further selective reporting or biases, and all of them are considered to have low risk of bias.

### Tests for bias

**Fig. 3:**
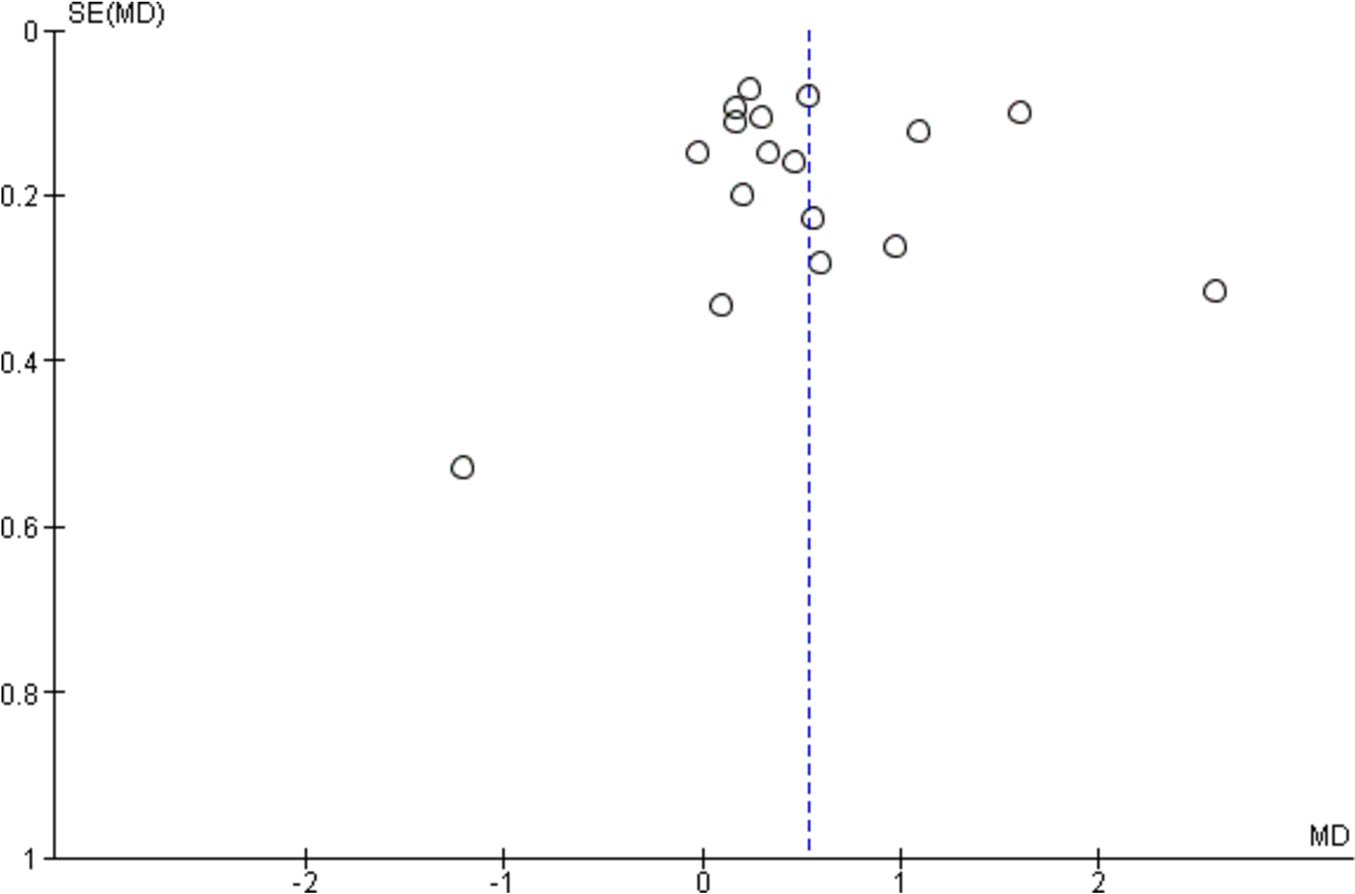
Bias funnel plot

This research includes outcome indicators for analysis, and the funnel plot demonstrates a distribution that is symmetrical, indicating the absence of publication bias, as seen in the Fig. 3.

### Efficacy tests

#### The relationship between teaching games in physical education and enjoyment of children and adolescents

**Fig. 4:**
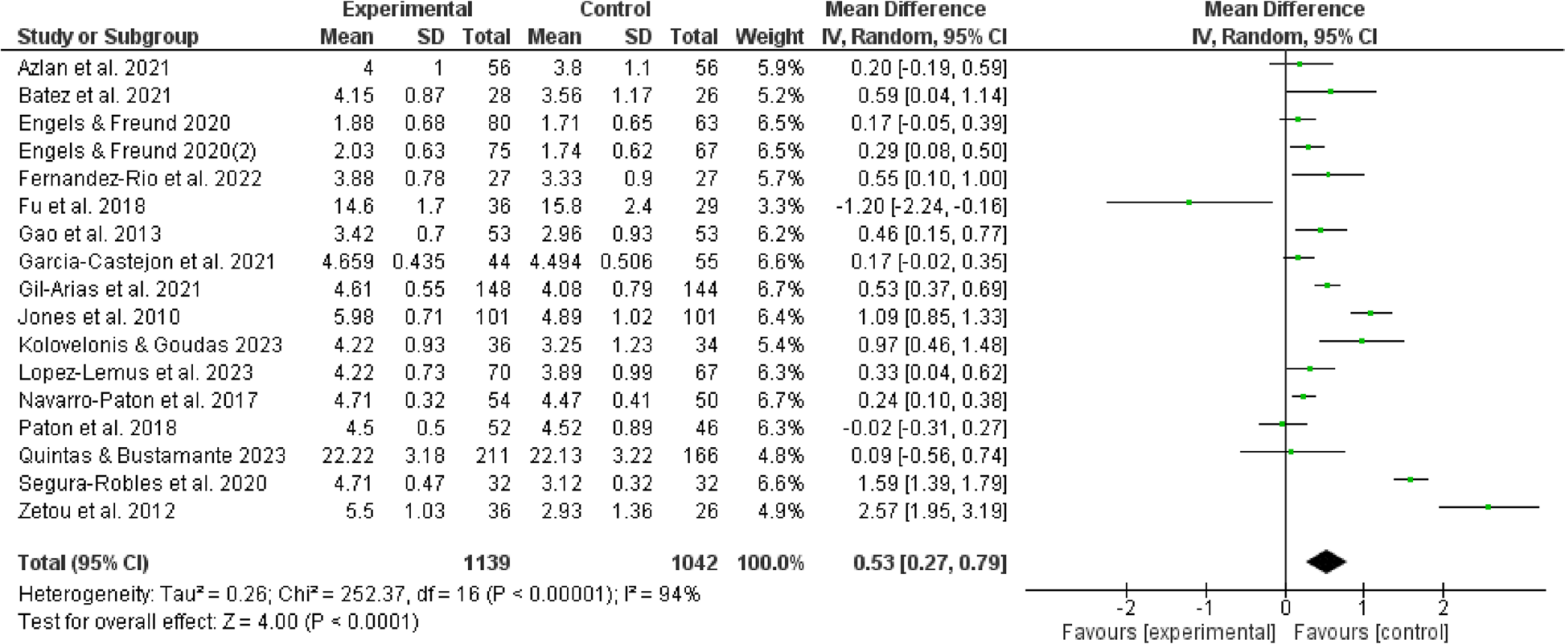
Forest plot depicting the relation between physical game teaching and enjoyment in children and adolescents

Heterogeneity tests were performed on the articles that were included in the analysis. Out of the total, 17 research (comprising 16 papers) indicated an altered association between the enjoyment experienced by children and adolescents in the context of teaching physical education games [1, 14, 18–30, 33]. The researchers apply a random effects model to collect the findings about the articles’ outcome indicators. This study includes 17 studies and 2181 participants in total, with 1042 in the control group and 1139 in the experimental group. The present research provides evidence supporting the favorable impact of a physical education intervention using games on the positive emotions of children and adolescents in the experimental group (MD = 0.53, 95% CI: [0.27, 0.79], *P* < 0.05), as depicted in Fig. 4.

#### Subgroup analyses

The combined impact size data for physical play teaching interventions on children and adolescents show a significant degree of variation. It is achieved by analyzing subgroups based on gender, age, duration, frequency, and cycling as potential influencing factors.

**Fig. 5:**
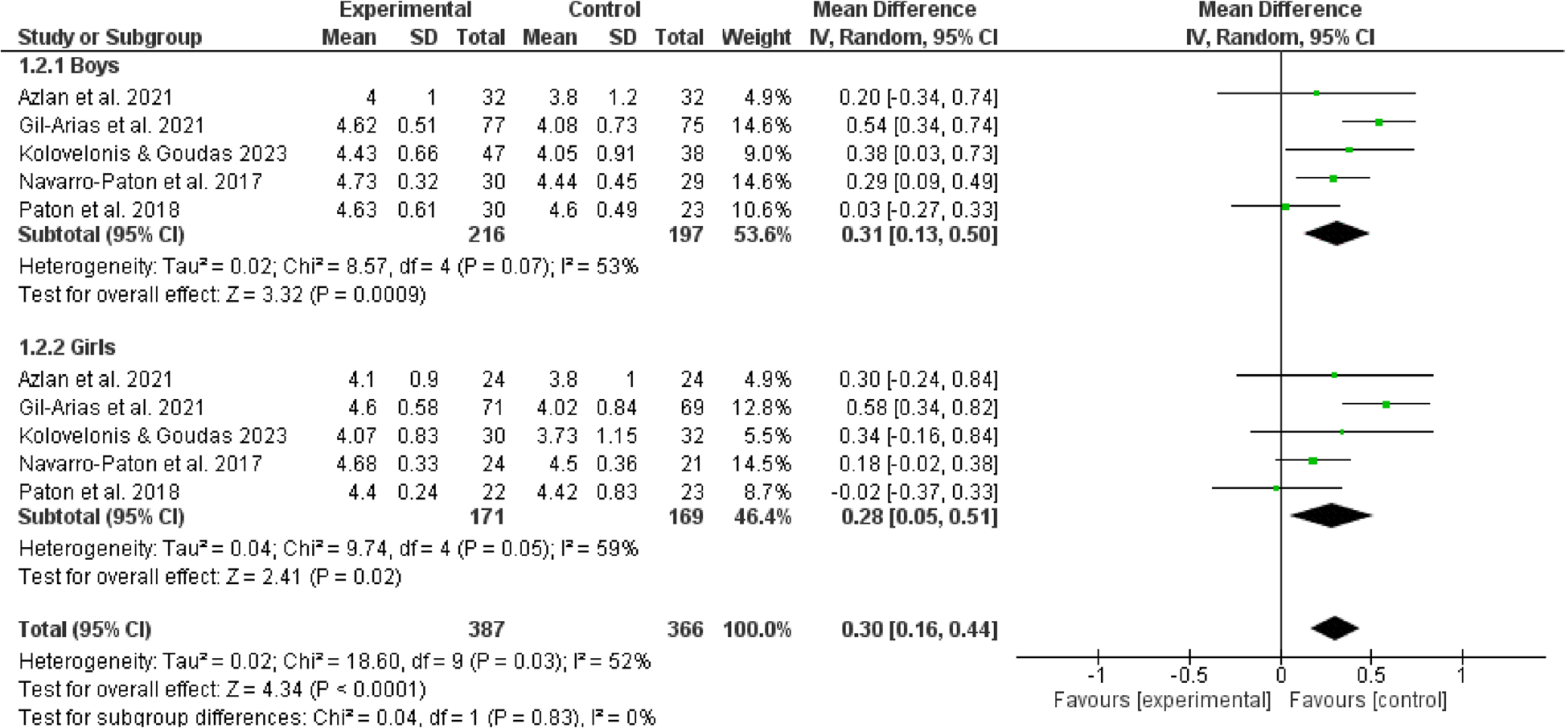
Forest plot depicting gender subgroup relationship between physical game teaching and enjoyment in children and adolescents

**Fig. 6:**
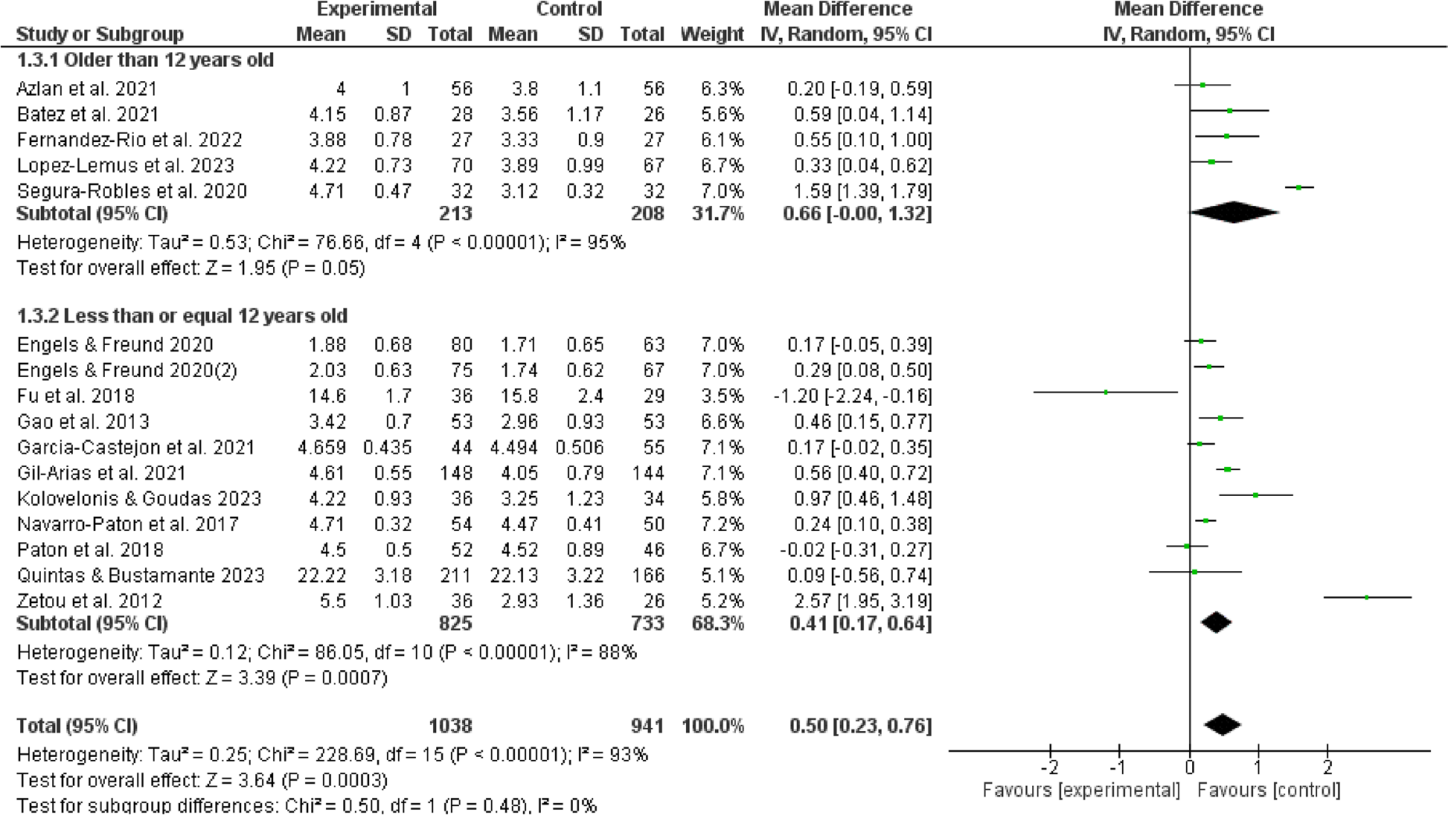
Forest plot depicting age subgroup relationship between physical game teaching and enjoyment in children and adolescents

**Fig. 7:**
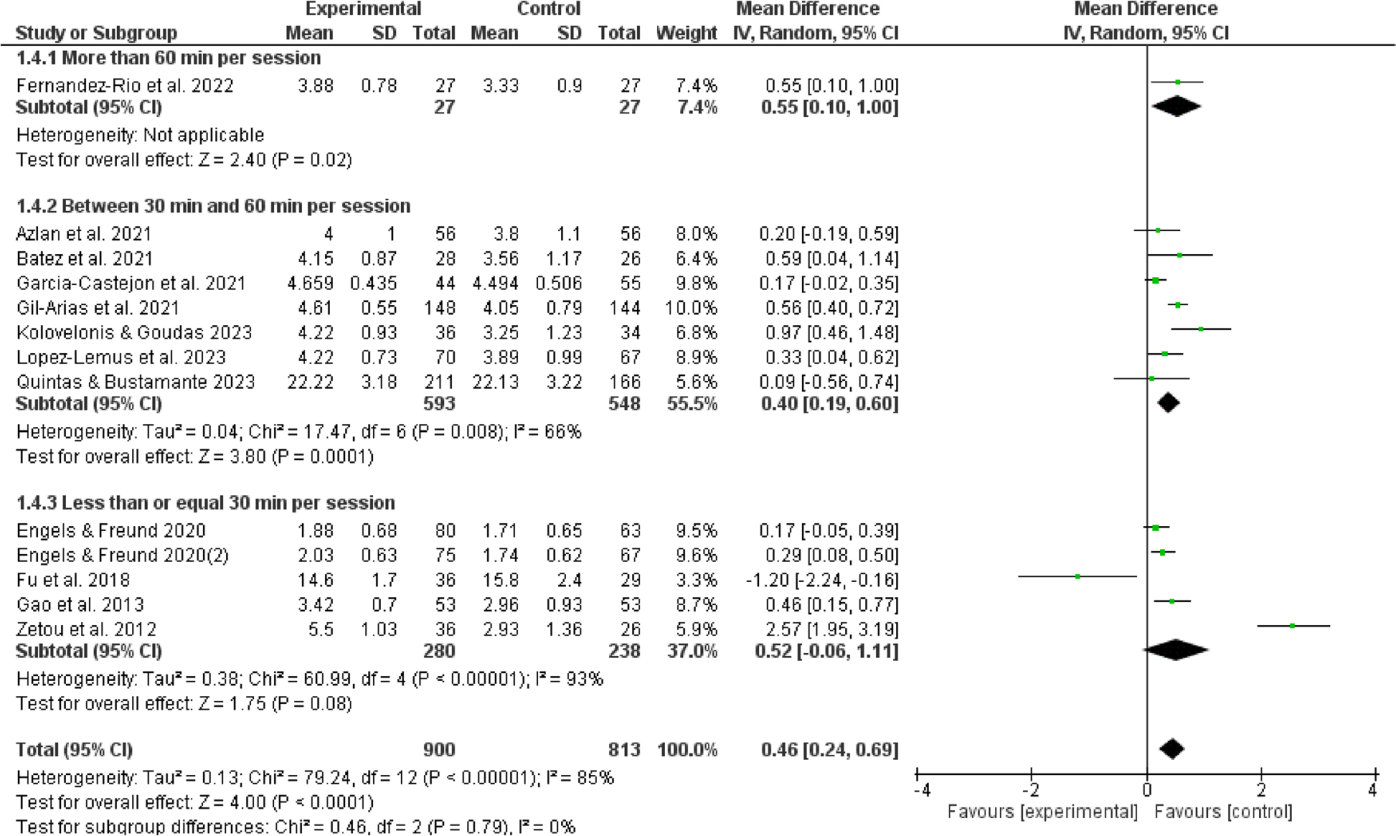
Forest plot depicting duration subgroup relationship between physical game teaching and enjoyment in children and adolescents

**Fig. 8:**
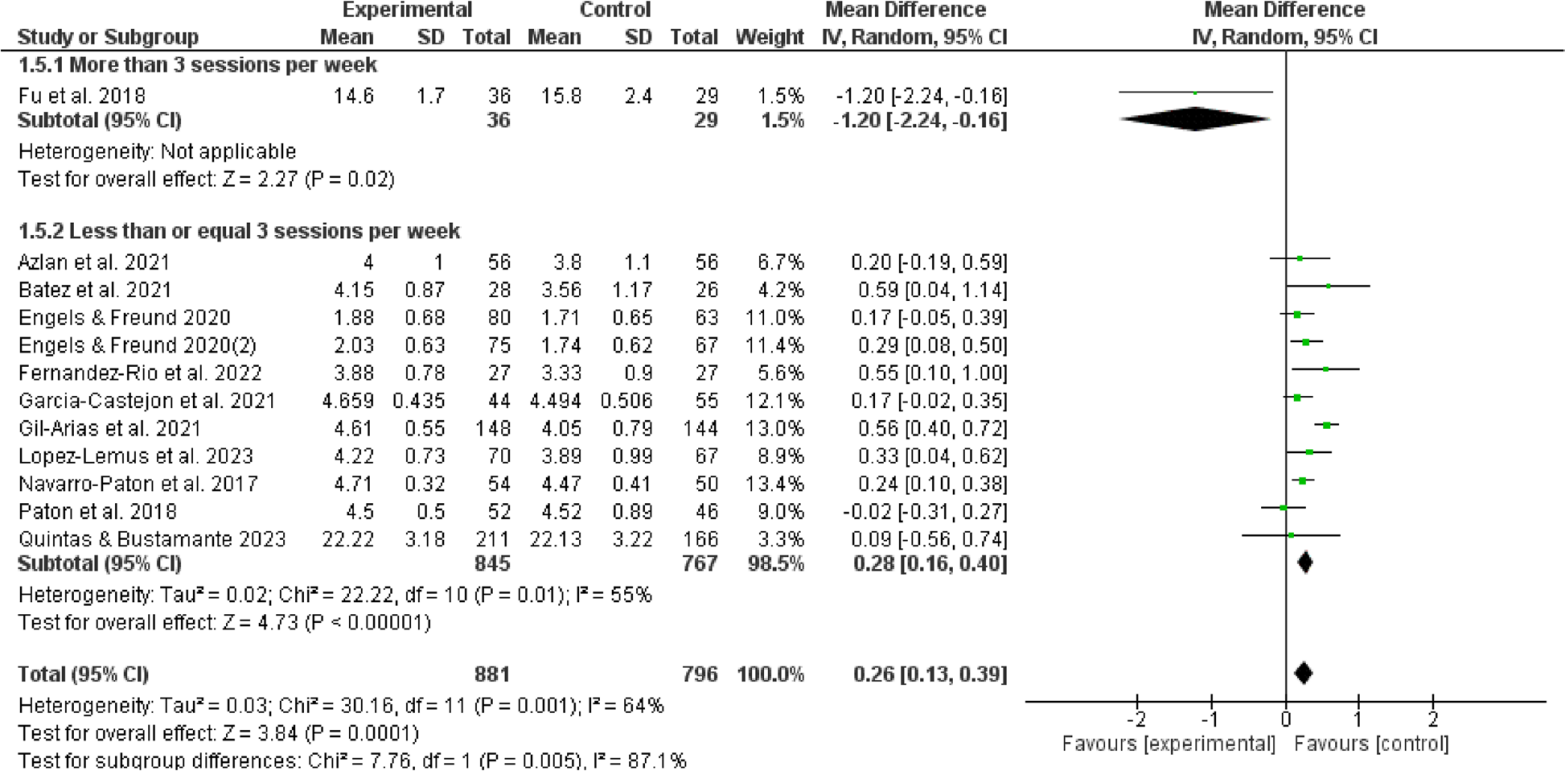
Forest plot depicting frequency subgroup relationship between physical game teaching and enjoyment in children and adolescents

**Fig. 9:**
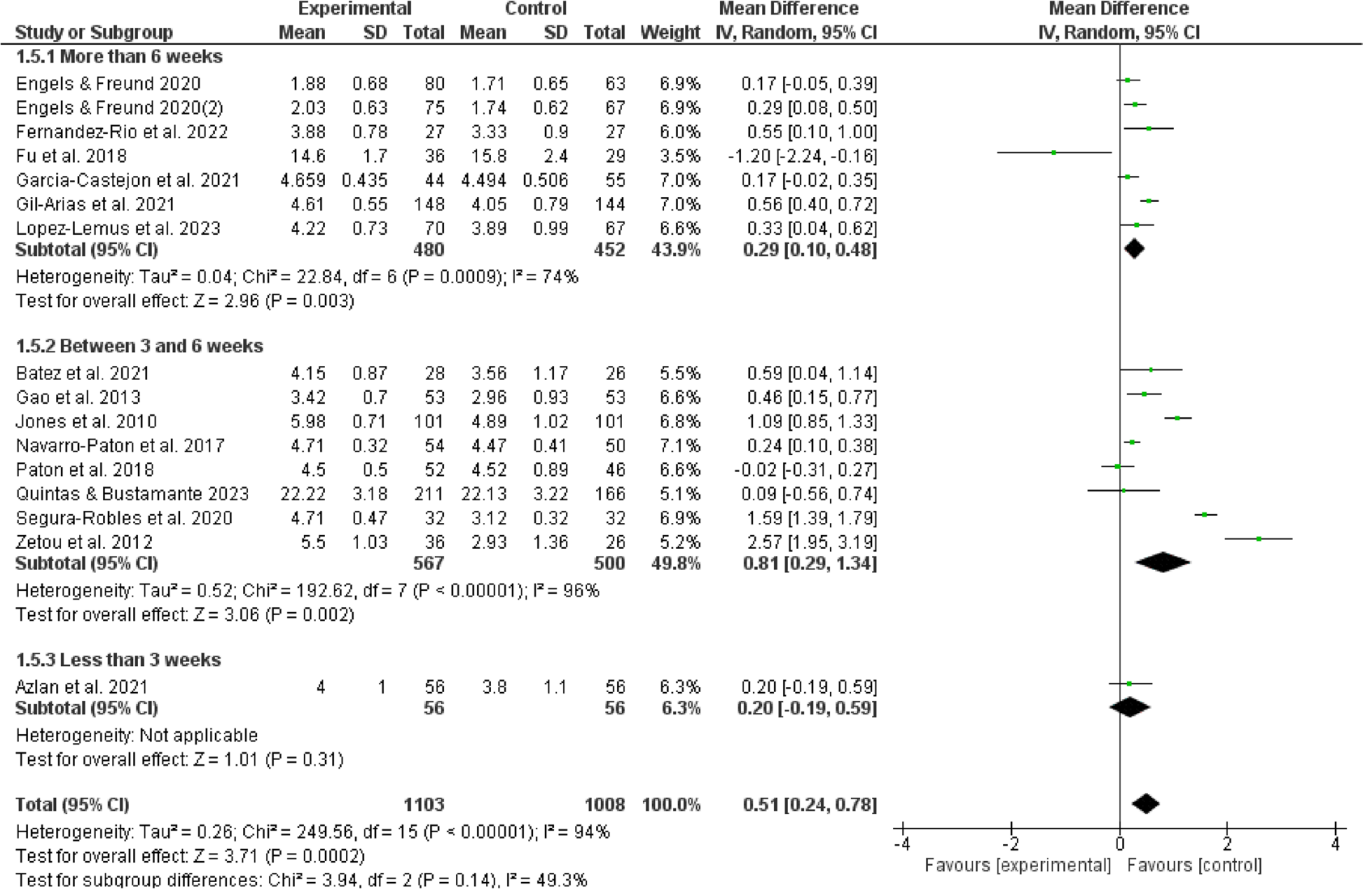
Forest plot depicting cycle subgroup relation between physical game teaching and enjoyment in children and adolescents

The results of subgroup analyses examining the influence of gender, age, duration, frequency, and cycling on the effects of games in physical education indicate that such games can enhance enjoyment of boys (MD = 0.31, 95% CI:[0.13,0.50], *P* < 0.05) and positively affect girls (MD = 0.28, 95%CI:[0.05,0.51], *P* < 0.05). Furthermore, it is observed that children aged 12 experienced an increasing enjoyment (MD = 0.41, 95% CI:[0.17,0.64], *P* < 0.05), whereas adolescents aged 12 and above do not show a similar increase (*P* > 0.05). The duration of physical education sessions ranging from 30 to 60 minutes (MD = 0.40,95%CI:[0.19,0.60], *P* < 0.05) can provide a favorable impact on enjoyment experienced by children and adolescents. Moreover, extending the duration of physical education beyond 60 minutes (MD = 0.55,95%CI:[0.10,1.00], *P* < 0.05) may also improve their enjoyment. However, noticeably, durations shorter than 30 minutes do not show the same good effect (*P* > 0.05). It is more feasible to provide physical game teaching within a frequency range of 1 to 3 sessions per week (MD = 0.28,95%CI:[0.16,0.40], *P* < 0.05) to elicit enjoyment among children and adolescents. Conversely, it is unsuitable to give physical game instructions, exceeding the threshold of 3 sessions per week (*P* > 0.05). The optimal duration for physical game teaching to elicit enjoyable outcomes in children and adolescents is between 3 to 6 weeks (MD = 0.81, 95%CI:[0.29,1.34],*P* < 0.05), but durations beyond 6 weeks are also considered acceptable (MD = 0.29, 95%CI:[0.10,0.48],*P* < 0.05). In contrast, it is not a proper option to be engaged in physical games for less than 3 weeks (*P* > 0.05). Hence, such factors as gender, age, duration, frequency, and cycle contribute significantly to the observed variation in satisfaction, as seen in Figs. 5, 6, 7, 8, 9.

## Discussion

### Main research analyses

The results of this study, including the analysis of 17 studies, show that the adoption of physical education game-based intervention has a beneficial effect on the enjoyment levels of children and adolescents. Such corresponds to the idea offered by Tornero and Capella, 2017, which claims that playing games adjusts to the emotional state of children and adolescents [34]. This advantageous feeling state can further improve their engagement in school sports activities [35, 36]. Physiological studies prove that engaging in physical activity or exercise causes the release of endorphins from the pituitary gland and subthalamus. Endorphins are hormones that induce feelings of calmness and pleasure, enhancing mood and creating an enjoyable experience for children and adolescents during physical education programs, including games [37–39]. Furthermore, when it comes to content, the teaching of physical education games appears to enhance the enjoyment experienced by children and adolescents to a greater extent than the classes of traditional physical education. In their study, Batez et al. (2021) discovered that adolescents in the experimental group who participated in the Teaching Games for Understanding (mini-volleyball) way experienced a greater sense of satisfaction compared to the control group during the post-test phase [23]. Lopez-Lemus et al. (2023) noticed that analyzing the pre-test and post-test results of both the experimental and control groups revealed that 67 students who were part of the Sport Education (SE)/Teaching for understanding (TGfU) experimental group, specifically focusing on handball, revealed enhancements in-game performance, enjoyment, perceptual skills, and intentions [21]. Similarly, researches on dance movement games and basketball games show superior levels of enjoyment compared to traditional teaching methods [19, 40]. Hence, this research posits that including games into physical education courses may effectively enhance the enjoyment of children and adolescents, making it a recommended approach compared to programmes that do not use games.

### Gender analysis

This study claims that practicing physical sports might affect enjoyment among individuals of different genders, with boys expressing a greater chance of experiencing enjoyment compared to girls. Research has shown that as they get older boys and girls display unique preferences. In a cultural analysis conducted by Joseph et al., 25 African American women were surveyed regarding their engagement in physical activities. The majority of these women reported positive and enjoyable experiences in childhood, but their feelings were not apparent during their youth [41]. Additionally, female adolescents had a lower frequency of pleasurable meets in physical exercise compared to male adolescents, and they also displayed negative emotions towards engaging in physical activity [42]. Even so, variations in the level of enjoyment based on gender are likely to be impacted by different types of sports games. Girls have a preference for cooperative activities, particularly dancing games [43], whereas boys seem to choose competitive fitness games [44]. In all, both males and females can experience enjoyment in physical education games, still, variations in the level of enjoyment may arise due to factors such as age and the specific type of game. It seems that gender alone is not the sole determinant of enjoyment, and further study is required to identify other contributing factors.

### Analysis of age

According to this study, teaching physical education games has a major effect on the enjoyable feelings of children below the age of 12. And yet, it does not have a substantial influence on teenagers aged 12 and above. In the opinion of Velez & Garcia, children between the ages of 9 and 12 have better levels of individual feelings of happiness compared to teenagers aged 13 to 17 [45]. Play is an essential element in the development of children’s motor skills and is intrinsically linked to enjoyment, which serves as a motivation for children to engage in physical exercise [46]. According to Bremer et al., a study demonstrated that children between the ages of 6 and 13 with autism who enjoyed their physical education sessions were more likely to engage in other physical activities [47]. Academic competition at school is an important factor that hinders the development of enjoyable feelings in teens, this is mostly caused by the negative effects of stress-induced depression and anxiety [48]. Mangerud found that engaging in physical exercise has an impact on the positive emotions of adolescents with anxiety disorders, including their enjoyment of sports circumstances [49]. As a result, teaching youngsters under the age of 12 physical sports proves to be a more successful method for obtaining enjoyment compared to teenagers aged 12 and above.

### Analysis of duration

The present research offers that applying a physical game lesson beyond a duration of 30 minutes has a favorable impact on the enjoyment of children and adolescents, but less than 30 minutes appears to have little to no effect. This corresponds to the findings of the Gil-Madrona, when children participated in 45 minutes of popular cooperative and cooperative-oppositional games [50]. Physical exercise in children and teenagers increases the release of neurotransmitters including dopamine and (−)-norepinephrine. These substances help to decrease depression and anxiety, leading to increased feelings of euphoria, achievement, and overall well-being, which improve over time [51–53]. Previous research has shown that children tend to get pleasure from short periods of intense physical activity followed by times of relaxation (similar to outside play), whereas adults may have a preference for lengthier activities [54]. However, Tobin et al. carried out experiments with participants of varying durations and determined that a 12-minute length of time was considered insufficient for players to become fully engaged in the game, thus serving merely as a warm-up period [55]. In addition, another study corroborated these findings by establishing that children exhibited diminished motivation and failed to experience enhanced enjoyment when engaging in sports games for a brief duration of 20 minutes [56]. In the end, engaging in sports games for no less than 30 minutes can lead to improved outcomes and heightened enjoyment for children and adolescents.

### Analysis of frequency

The analyses suggested that a teaching intervention based on physical education and games held 1 to 3 times per week is suitable for children and adolescents to experience enjoyment. However, doing more than 3 sessions per week seems unsuitable. Studies indicate a correlation between the frequency of participating in physical activity and experiencing positive emotions [57]. Furthermore, sustaining a proper frequency of physical activity could promote the feeling of Feelings of happiness. For instance, Batez and Gil-Arias both applied the teaching games for understanding (TGfU) approach in a physical education program, results indicated that students’ level of enjoyment somewhat improved when the games were taught twice weekly [23, 58]. However, excessive participation in game activities without sufficient time for rest and recovery can lead to the build-up of lactic acid in the muscles, resulting in increased physical fatigue and negatively impacting the individual’s mood, finally diminishing the enjoyment of the gaming experience [59–62]. Temporary breaks can effectively facilitate physical recovery during physical education games, it not only promotes bodily rejuvenation but also enhances the enjoyment of children and adolescents [63]. Therefore, it is advisable to offer 1–3 lessons per week to optimise the teaching of physical education games.

### Analysis of cycles

This study stated that physical education game teaching interventions lasting between 3 to 6 weeks and more than 6 weeks are ideal for improving the enjoyable outcomes of children and adolescents. Conversely, interventions lasting less than 3 weeks are not advisable. This conclusion is supported by the findings of previous studies. Some curriculum interventions like Zetou et al. designed a 4-week ‘Play and Stay’ tennis teaching programme, Jones et al.’s 6-week Teaching Games for Understanding and Fernandez-Rio et al.’s 9-week Gamification [22, 24, 26]. Findings show an increase in students’ enjoyment, linked to the regular meeting of their intrinsic drive in the physical education classroom. Several studies indicated that short physical procedures lasting only 1 week do not effectively assess the intrinsic motivation of participants [64, 65], thus posing difficulties with stimulating the generation of enjoyable sensations in persons. Moreover, extended periods of physical play may result in decreased intrinsic motivation or boredom in children and adolescents [66, 67]. Fu et al. and Zeng et al. propose that while physical play at first brings joy, enjoyment diminishes over time [33, 68]. In conclusion, children and adolescents should engage in playful activities for a minimum of 3 weeks, while also ensuring that the play program offers a variety of activities and rich content to enhance their enjoyment.

## Conclusion

This study applies a meta-analysis to examine the significance of teaching games in physical education regarding emotional delight experienced by children and adolescents. Gender, age, duration, frequency and cycles may be the reasons for variances impacting the research outcomes. This research finds that male participants are more likely to show enjoyable behavior compared with their female counterparts as for games teaching in physical courses. However, it should be noted that gender disparities may be influenced by variables like age and the specific kind of sports taught in class. Besides,Children engage in a minimum of 30 minutes every session, attending 1 to 3 sessions per week, so guaranteeing that the physical education and games curriculum is delivered for a span exceeding 3 weeks. This approach aims to foster positive affective experiences among children, thereby facilitating the attainment of optimum outcomes.

## Limitations and future research

Apart from the meaningful findings, this research also has some drawbacks. Firstly, it adopts a meta-analytical approach to examine the influence of games teaching in physical education on enjoyment in children and adolescent. It primarily focuses on the outcomes of curriculum and teaching implementation. Consequently, the results may not be applicable to other contexts, such as after-school physical game activities, community physical game activities, and family physical game activities. Furthermore, the 17 studies analyzed in this research have insufficient data on duration, frequency, and period. This insufficient information may influence the statistical accuracy of conducting effect size tests. Additionally, the 17 studies fail to offer any data about the intensity of the activities employed in games teaching in physical education, such as heart rate, oxygen uptake, and respiratory rate. Consequently, future studies can address this gap in knowledge. Additionally, the current research does not ascertain the ideal upper threshold for the duration of engagement in the activity. This aspect warrants further exploration in a later literature review. Additionally, it is crucial to evaluate other variables which may influence the research outcomes, such as the specific nature of the sports game being analyzed. For further study, it would be fruitful to classify different sorts of sports games to improve the whole quality of the research.

## Data Availability

All data generated or analysed during this study are included in this published article.
